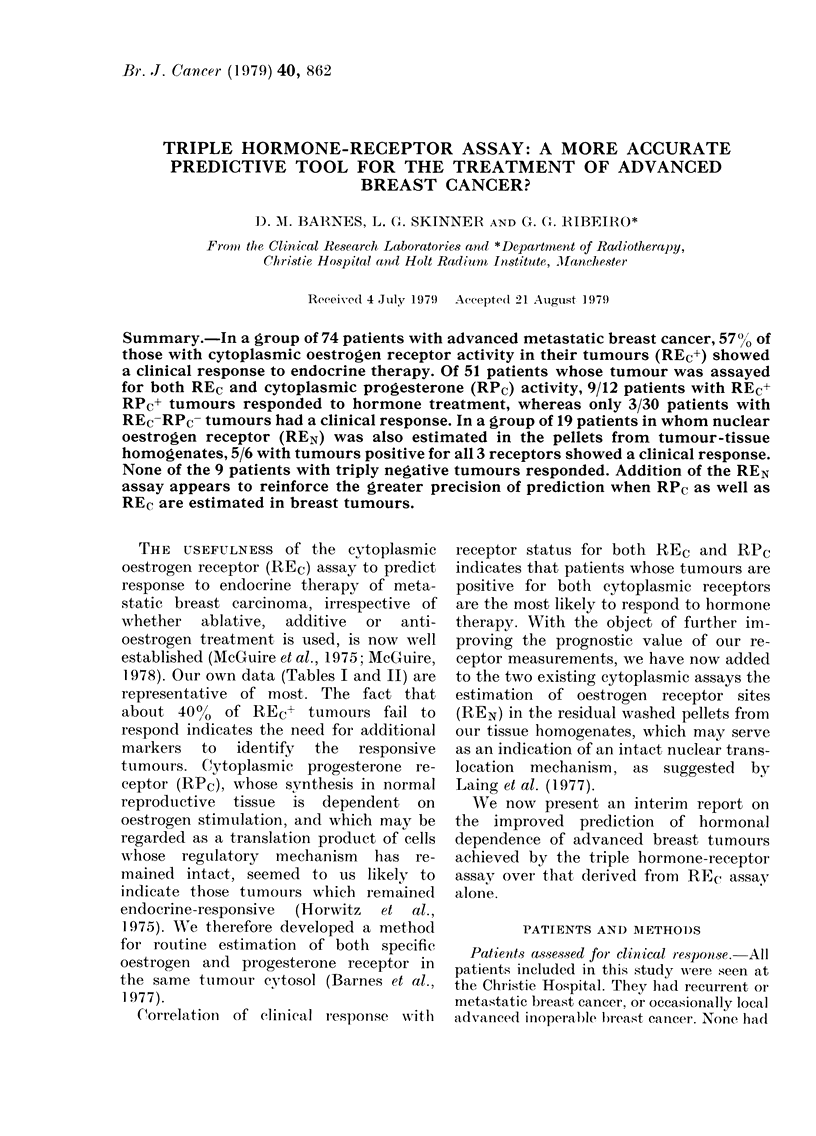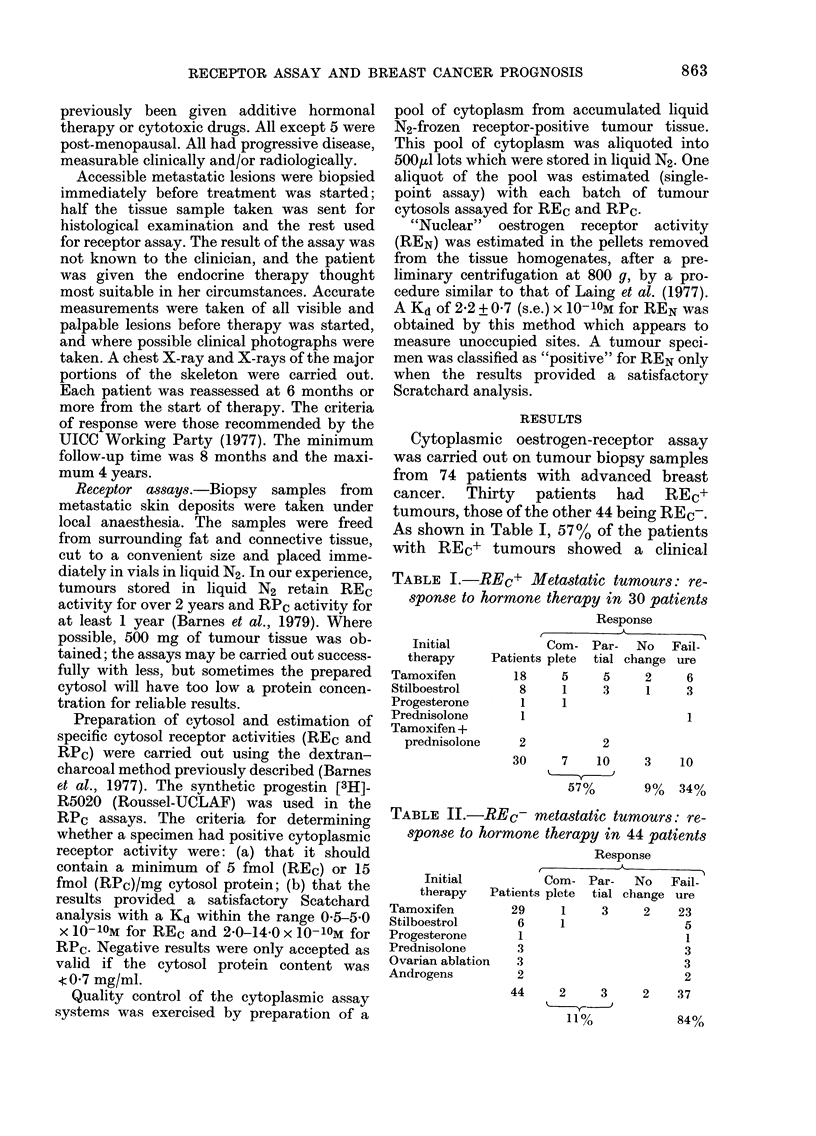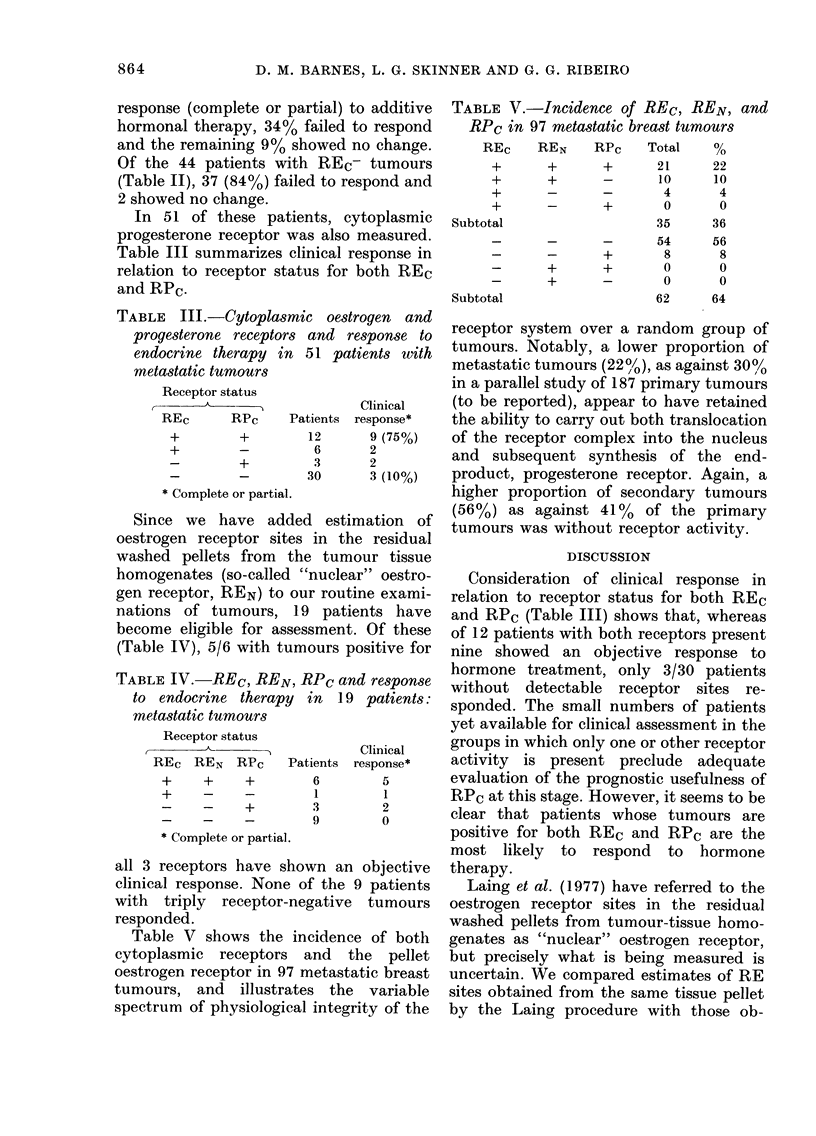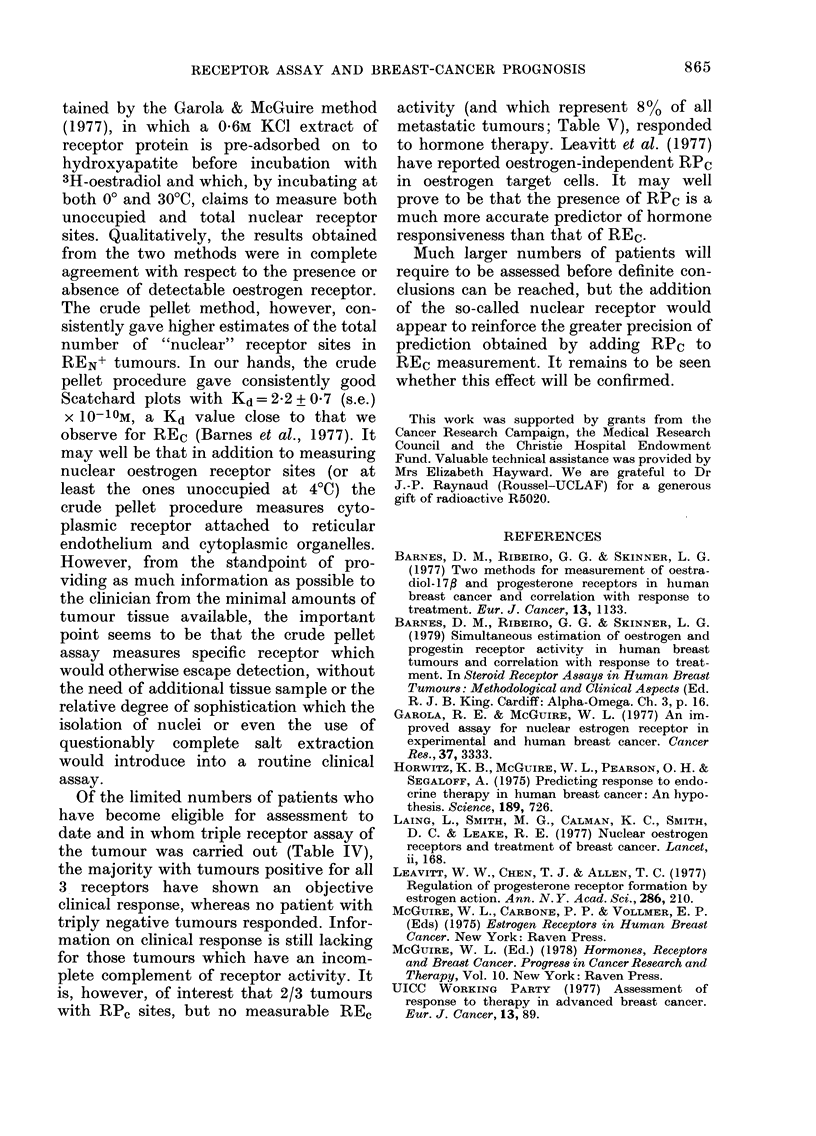# Triple hormone-receptor assay: a more accurate predictive tool for the treatment of advanced breast cancer?

**DOI:** 10.1038/bjc.1979.277

**Published:** 1979-12

**Authors:** D. M. Barnes, L. G. Skinner, G. G. Ribeiro

## Abstract

In a group of 74 patients with advanced metastatic breast cancer, 57% of those with cytoplasmic oestrogen receptor activity in their tumours (REC+) showed a clinical response to endocrine therapy. Of 51 patients whose tumour was assayed for both REC and cytoplasmic progesterone (RPC) activity, 9/12 patients with REC+ RPC+ tumours responded to hormone treatment, whereas only 3/30 patients with REC-RPC-tumours had a clinical response. In a group of 19 patients in whom nuclear oestrogen receptor (REN) was also estimated in the pellets from tumour-tissue homogenates, 5/6 with tumours positive for all 3 receptors showed a clinical response. None of the 9 patients with triply negative tumours responded. Addition of the REN assay appears to reinforce the greater precision of prediction when RPC as well as REC are estimated in breast tumours.


*Br. J. Cancer* (1979) **40**, 862

### TRIPLE HORMONE-RECEPTOR ASSAY: A MORE ACCURATE PREDICTIVE TOOL FOR THE TREATMENT OF ADVANCED BREAST CANCER?

D. M. BARNES, L. G. SKINNER AND G. G. RIBEIRO*

*From the Clinical Research Laboratories and *Department of Radiotherapy, Christie Hospital and Holt Radium Institute, Manchester*

Received 4 July 1979 Accepted 21 August 1979

**Summary.—In a group of 74 patients with advanced metastatic breast cancer, 57% of those with cytoplasmic oestrogen receptor activity in their tumours ($RE_C^+$) showed a clinical response to endocrine therapy. Of 51 patients whose tumour was assayed for both $RE_C$ and cytoplasmic progesterone ($RP_C$) activity, 9/12 patients with $RE_C^+$ $RP_C^+$ tumours responded to hormone treatment, whereas only 3/30 patients with $RE_C^-RP_C^-$ tumours had a clinical response. In a group of 19 patients in whom nuclear oestrogen receptor ($RE_N$) was also estimated in the pellets from tumour-tissue homogenates, 5/6 with tumours positive for all 3 receptors showed a clinical response. None of the 9 patients with triply negative tumours responded. Addition of the $RE_N$ assay appears to reinforce the greater precision of prediction when $RP_C$ as well as $RE_C$ are estimated in breast tumours.**

THE USEFULNESS of the cytoplasmic oestrogen receptor ($RE_C$) assay to predict response to endocrine therapy of metastatic breast carcinoma, irrespective of whether ablative, additive or anti-oestrogen treatment is used, is now well established (McGuire *et al.*, 1975; McGuire, 1978). Our own data (Tables I and II) are representative of most. The fact that about 40% of $RE_C^+$ tumours fail to respond indicates the need for additional markers to identify the responsive tumours. Cytoplasmic progesterone receptor ($RP_C$), whose synthesis in normal reproductive tissue is dependent on oestrogen stimulation, and which may be regarded as a translation product of cells whose regulatory mechanism has remained intact, seemed to us likely to indicate those tumours which remained endocrine-responsive (Horwitz *et al.*, 1975). We therefore developed a method for routine estimation of both specific oestrogen and progesterone receptor in the same tumour cytosol (Barnes *et al.*, 1977).

Correlation of clinical response with receptor status for both $RE_C$ and $RP_C$ indicates that patients whose tumours are positive for both cytoplasmic receptors are the most likely to respond to hormone therapy. With the object of further improving the prognostic value of our receptor measurements, we have now added to the two existing cytoplasmic assays the estimation of oestrogen receptor sites ($RE_N$) in the residual washed pellets from our tissue homogenates, which may serve as an indication of an intact nuclear translocation mechanism, as suggested by Laing *et al.* (1977).

We now present an interim report on the improved prediction of hormonal dependence of advanced breast tumours achieved by the triple hormone-receptor assay over that derived from $RE_C$ assay alone.

### PATIENTS AND METHODS

*Patients assessed for clinical response.*—All patients included in this study were seen at the Christie Hospital. They had recurrent or metastatic breast cancer, or occasionally local advanced inoperable breast cancer. None had previously been given additive hormonal therapy or cytotoxic drugs. All except 5 were post-menopausal. All had progressive disease, measurable clinically and/or radiologically.

Accessible metastatic lesions were biopsied immediately before treatment was started; half the tissue sample taken was sent for histological examination and the rest used for receptor assay. The result of the assay was not known to the clinician, and the patient was given the endocrine therapy thought most suitable in her circumstances. Accurate measurements were taken of all visible and palpable lesions before therapy was started, and where possible clinical photographs were taken. A chest X-ray and X-rays of the major portions of the skeleton were carried out. Each patient was reassessed at 6 months or more from the start of therapy. The criteria of response were those recommended by the UICC Working Party (1977). The minimum follow-up time was 8 months and the maximum 4 years.

*Receptor assays.*—Biopsy samples from metastatic skin deposits were taken under local anaesthesia. The samples were freed from surrounding fat and connective tissue, cut to a convenient size and placed immediately in vials in liquid $N_2$. In our experience, tumours stored in liquid $N_2$ retain $RE_C$ activity for over 2 years and $RP_C$ activity for at least 1 year (Barnes *et al.*, 1979). Where possible, 500 mg of tumour tissue was obtained; the assays may be carried out successfully with less, but sometimes the prepared cytosol will have too low a protein concentration for reliable results.

Preparation of cytosol and estimation of specific cytosol receptor activities ($RE_C$ and $RP_C$) were carried out using the dextran–charcoal method previously described (Barnes *et al.*, 1977). The synthetic progestin [$^3$H]-R5020 (Roussel-UCLAF) was used in the $RP_C$ assays. The criteria for determining whether a specimen had positive cytoplasmic receptor activity were: (a) that it should contain a minimum of 5 fmol ($RE_C$) or 15 fmol ($RP_C$)/mg cytosol protein; (b) that the results provided a satisfactory Scatchard analysis with a $K_d$ within the range 0·5–5·0 $\times 10^{-10}$M for $RE_C$ and 2·0–14·0 $\times 10^{-10}$M for $RP_C$. Negative results were only accepted as valid if the cytosol protein content was $\nless$ 0·7 mg/ml.

Quality control of the cytoplasmic assay systems was exercised by preparation of a pool of cytoplasm from accumulated liquid $N_2$-frozen receptor-positive tumour tissue. This pool of cytoplasm was aliquoted into 500$\mu$l lots which were stored in liquid $N_2$. One aliquot of the pool was estimated (single-point assay) with each batch of tumour cytosols assayed for $RE_C$ and $RP_C$.

"Nuclear" oestrogen receptor activity ($RE_N$) was estimated in the pellets removed from the tissue homogenates, after a preliminary centrifugation at 800 *g*, by a procedure similar to that of Laing *et al.* (1977). A $K_d$ of 2·2 ± 0·7 (s.e.) $\times 10^{-10}$M for $RE_N$ was obtained by this method which appears to measure unoccupied sites. A tumour specimen was classified as "positive" for $RE_N$ only when the results provided a satisfactory Scatchard analysis.

### RESULTS

Cytoplasmic oestrogen-receptor assay was carried out on tumour biopsy samples from 74 patients with advanced breast cancer. Thirty patients had $RE_C^+$ tumours, those of the other 44 being $RE_C^-$. As shown in Table I, 57% of the patients with $RE_C^+$ tumours showed a clinical

TABLE I.—*$RE_C^+$ Metastatic tumours: response to hormone therapy in* 30 *patients*

| Initial therapy | Patients | Response: Complete | Partial | No change | Failure |
|---|---|---|---|---|---|
| Tamoxifen | 18 | 5 | 5 | 2 | 6 |
| Stilboestrol | 8 | 1 | 3 | 1 | 3 |
| Progesterone | 1 | 1 | | | |
| Prednisolone | 1 | | | | 1 |
| Tamoxifen + prednisolone | 2 | | 2 | | |
| | 30 | 7 | 10 | 3 | 10 |
| | | 57% | | 9% | 34% |

TABLE II.—*$RE_C^-$ metastatic tumours: response to hormone therapy in* 44 *patients*

| Initial therapy | Patients | Response: Complete | Partial | No change | Failure |
|---|---|---|---|---|---|
| Tamoxifen | 29 | 1 | 3 | 2 | 23 |
| Stilboestrol | 6 | 1 | | | 5 |
| Progesterone | 1 | | | | 1 |
| Prednisolone | 3 | | | | 3 |
| Ovarian ablation | 3 | | | | 3 |
| Androgens | 2 | | | | 2 |
| | 44 | 2 | 3 | 2 | 37 |
| | | 11% | | | 84% |

response (complete or partial) to additive hormonal therapy, 34% failed to respond and the remaining 9% showed no change. Of the 44 patients with $RE_C^-$ tumours (Table II), 37 (84%) failed to respond and 2 showed no change.

In 51 of these patients, cytoplasmic progesterone receptor was also measured. Table III summarizes clinical response in relation to receptor status for both $RE_C$ and $RP_C$.

TABLE III.—*Cytoplasmic oestrogen and progesterone receptors and response to endocrine therapy in* 51 *patients with metastatic tumours*

| Receptor status | | | |
|---|---|---|---|
| $RE_C$ | $RP_C$ | Patients | Clinical response* |
| + | + | 12 | 9 (75%) |
| + | − | 6 | 2 |
| − | + | 3 | 2 |
| − | − | 30 | 3 (10%) |

* Complete or partial.

Since we have added estimation of oestrogen receptor sites in the residual washed pellets from the tumour tissue homogenates (so-called "nuclear" oestrogen receptor, $RE_N$) to our routine examinations of tumours, 19 patients have become eligible for assessment. Of these (Table IV), 5/6 with tumours positive for all 3 receptors have shown an objective clinical response. None of the 9 patients with triply receptor-negative tumours responded.

TABLE IV.—*$RE_C$, $RE_N$, $RP_C$ and response to endocrine therapy in* 19 *patients: metastatic tumours*

| Receptor status | | | | |
|---|---|---|---|---|
| $RE_C$ | $RE_N$ | $RP_C$ | Patients | Clinical response* |
| + | + | + | 6 | 5 |
| + | − | − | 1 | 1 |
| − | − | + | 3 | 2 |
| − | − | − | 9 | 0 |

* Complete or partial.

Table V shows the incidence of both cytoplasmic receptors and the pellet oestrogen receptor in 97 metastatic breast tumours, and illustrates the variable spectrum of physiological integrity of the receptor system over a random group of tumours. Notably, a lower proportion of metastatic tumours (22%), as against 30% in a parallel study of 187 primary tumours (to be reported), appear to have retained the ability to carry out both translocation of the receptor complex into the nucleus and subsequent synthesis of the end-product, progesterone receptor. Again, a higher proportion of secondary tumours (56%) as against 41% of the primary tumours was without receptor activity.

TABLE V.—*Incidence of $RE_C$, $RE_N$, and $RP_C$ in* 97 *metastatic breast tumours*

| $RE_C$ | $RE_N$ | $RP_C$ | Total | % |
|---|---|---|---|---|
| + | + | + | 21 | 22 |
| + | + | − | 10 | 10 |
| + | − | − | 4 | 4 |
| + | − | + | 0 | 0 |
| Subtotal | | | 35 | 36 |
| − | − | − | 54 | 56 |
| − | − | + | 8 | 8 |
| − | + | + | 0 | 0 |
| − | + | − | 0 | 0 |
| Subtotal | | | 62 | 64 |

DISCUSSION

Consideration of clinical response in relation to receptor status for both $RE_C$ and $RP_C$ (Table III) shows that, whereas of 12 patients with both receptors present nine showed an objective response to hormone treatment, only 3/30 patients without detectable receptor sites responded. The small numbers of patients yet available for clinical assessment in the groups in which only one or other receptor activity is present preclude adequate evaluation of the prognostic usefulness of $RP_C$ at this stage. However, it seems to be clear that patients whose tumours are positive for both $RE_C$ and $RP_C$ are the most likely to respond to hormone therapy.

Laing *et al.* (1977) have referred to the oestrogen receptor sites in the residual washed pellets from tumour-tissue homogenates as "nuclear" oestrogen receptor, but precisely what is being measured is uncertain. We compared estimates of RE sites obtained from the same tissue pellet by the Laing procedure with those obtained by the Garola & McGuire method (1977), in which a 0·6M KCl extract of receptor protein is pre-adsorbed on to hydroxyapatite before incubation with $^{3}$H-oestradiol and which, by incubating at both 0° and 30°C, claims to measure both unoccupied and total nuclear receptor sites. Qualitatively, the results obtained from the two methods were in complete agreement with respect to the presence or absence of detectable oestrogen receptor. The crude pellet method, however, consistently gave higher estimates of the total number of "nuclear" receptor sites in $RE_N^+$ tumours. In our hands, the crude pellet procedure gave consistently good Scatchard plots with $K_d = 2 \cdot 2 \pm 0 \cdot 7$ (s.e.) $\times 10^{-10}$M, a $K_d$ value close to that we observe for $RE_C$ (Barnes *et al.*, 1977). It may well be that in addition to measuring nuclear oestrogen receptor sites (or at least the ones unoccupied at 4°C) the crude pellet procedure measures cytoplasmic receptor attached to reticular endothelium and cytoplasmic organelles. However, from the standpoint of providing as much information as possible to the clinician from the minimal amounts of tumour tissue available, the important point seems to be that the crude pellet assay measures specific receptor which would otherwise escape detection, without the need of additional tissue sample or the relative degree of sophistication which the isolation of nuclei or even the use of questionably complete salt extraction would introduce into a routine clinical assay.

Of the limited numbers of patients who have become eligible for assessment to date and in whom triple receptor assay of the tumour was carried out (Table IV), the majority with tumours positive for all 3 receptors have shown an objective clinical response, whereas no patient with triply negative tumours responded. Information on clinical response is still lacking for those tumours which have an incomplete complement of receptor activity. It is, however, of interest that 2/3 tumours with $RP_c$ sites, but no measurable $RE_c$ activity (and which represent 8% of all metastatic tumours; Table V), responded to hormone therapy. Leavitt *et al.* (1977) have reported oestrogen-independent $RP_C$ in oestrogen target cells. It may well prove to be that the presence of $RP_C$ is a much more accurate predictor of hormone responsiveness than that of $RE_C$.

Much larger numbers of patients will require to be assessed before definite conclusions can be reached, but the addition of the so-called nuclear receptor would appear to reinforce the greater precision of prediction obtained by adding $RP_C$ to $RE_C$ measurement. It remains to be seen whether this effect will be confirmed.

This work was supported by grants from the Cancer Research Campaign, the Medical Research Council and the Christie Hospital Endowment Fund. Valuable technical assistance was provided by Mrs Elizabeth Hayward. We are grateful to Dr J.-P. Raynaud (Roussel–UCLAF) for a generous gift of radioactive R5020.

### REFERENCES

BARNES, D. M., RIBEIRO, G. G. & SKINNER, L. G. (1977) Two methods for measurement of oestradiol-17β and progesterone receptors in human breast cancer and correlation with response to treatment. *Eur. J. Cancer*, **13**, 1133.

BARNES, D. M., RIBEIRO, G. G. & SKINNER, L. G. (1979) Simultaneous estimation of oestrogen and progestin receptor activity in human breast tumours and correlation with response to treatment. In *Steroid Receptor Assays in Human Breast Tumours: Methodological and Clinical Aspects* (Ed. R. J. B. King. Cardiff: Alpha-Omega. Ch. 3, p. 16.

GAROLA, R. E. & MCGUIRE, W. L. (1977) An improved assay for nuclear estrogen receptor in experimental and human breast cancer. *Cancer Res.*, **37**, 3333.

HORWITZ, K. B., MCGUIRE, W. L., PEARSON, O. H. & SEGALOFF, A. (1975) Predicting response to endocrine therapy in human breast cancer: An hypothesis. *Science*, **189**, 726.

LAING, L., SMITH, M. G., CALMAN, K. C., SMITH, D. C. & LEAKE, R. E. (1977) Nuclear oestrogen receptors and treatment of breast cancer. *Lancet*, ii, 168.

LEAVITT, W. W., CHEN, T. J. & ALLEN, T. C. (1977) Regulation of progesterone receptor formation by estrogen action. *Ann. N.Y. Acad. Sci.*, **286**, 210.

MCGUIRE, W. L., CARBONE, P. P. & VOLLMER, E. P. (Eds) (1975) *Estrogen Receptors in Human Breast Cancer*. New York: Raven Press.

MCGUIRE, W. L. (Ed.) (1978) *Hormones, Receptors and Breast Cancer. Progress in Cancer Research and Therapy*, Vol. 10. New York: Raven Press.

UICC WORKING PARTY (1977) Assessment of response to therapy in advanced breast cancer. *Eur. J. Cancer*, **13**, 89.